# Two-step verification method for Monte Carlo codes in biomedical optics applications

**DOI:** 10.1117/1.JBO.27.8.083018

**Published:** 2022-04-20

**Authors:** Angelo Sassaroli, Federico Tommasi, Stefano Cavalieri, Lorenzo Fini, André Liemert, Alwin Kienle, Tiziano Binzoni, Fabrizio Martelli

**Affiliations:** a Tufts University, Department of Biomedical Engineering, Medford, Massachusetts, United States; b Dipartimento di Fisica e Astronomia dell’Università degli Studi di Firenze, Sesto Fiorentino, Italy; c Institut für Lasertechnologien in der Medizin und Meßtechnik an der Universität Ulm (ILM), Ulm, Germany; d University of Geneva, Department of Basic Neurosciences, Geneva, Switzerland; e University Hospital, Department of Radiology and Medical Informatics, Geneva, Switzerland

**Keywords:** Monte Carlo method, biomedical optics, verification procedure, analytical benchmarks, forward solvers, radiative transfer equation

## Abstract

**Significance:**

Code verification is an unavoidable step prior to using a Monte Carlo (MC) code. Indeed, in biomedical optics, a widespread verification procedure for MC codes is still missing. Analytical benchmarks that can be easily used for the verification of different MC routines offer an important resource.

**Aim:**

We aim to provide a two-step verification procedure for MC codes enabling the two main tasks of an MC simulator: (1) the generation of photons’ trajectories and (2) the intersections of trajectories with boundaries separating the regions with different optical properties. The proposed method is purely based on elementary analytical benchmarks, therefore, the correctness of an MC code can be assessed with a one-sample t-test.

**Approach:**

The two-step verification is based on the following two analytical benchmarks: (1) the exact analytical formulas for the statistical moments of the spatial coordinates where the scattering events occur in an infinite medium and (2) the exact invariant solutions of the radiative transfer equation for radiance, fluence rate, and mean path length in media subjected to a Lambertian illumination.

**Results:**

We carried out a wide set of comparisons between MC results and the two analytical benchmarks for a wide range of optical properties (from non-scattering to highly scattering media, with different types of scattering functions) in an infinite non-absorbing medium (step 1) and in a non-absorbing slab (step 2). The deviations between MC results and exact analytical values are usually within two standard errors (i.e., t-tests not rejected at a 5% level of significance). The comparisons show that the accuracy of the verification increases with the number of simulated trajectories so that, in principle, an arbitrary accuracy can be obtained.

**Conclusions:**

Given the simplicity of the verification method proposed, we envision that it can be widely used in the field of biomedical optics.

## 1 Introduction

The verification of a Monte Carlo (MC) code is an important aspect of the whole process of confirmation that assures the scientific community of the reliability of its results.^1^ In the process of confirmation, we can distinguish between a verification phase and a validation phase. The verification of an MC code is typically done by comparisons between its results and those obtained either with analytical benchmarks,^1^^–^^3^ or more often with previously verified MC codes. In contrast, the validation of an MC code is done by comparisons between its results and those obtained with experiments.^4^^,^^5^ In this work, we are concerned only with the verification of an MC code. In the last decades, the modeling of heterogeneous tissue structures in MC codes for photon transport has required the development of algorithms with increasing complexity.^6^^–^^17^ Therefore, the need for a thorough verification procedure has become more and more urgent.

As a matter of fact, most of the MC codes developed in biomedical optics have been verified partially or exclusively by means of comparisons with previously verified codes.^6^^–^^18^ In the cross-verification procedures between MC results,^9^^–^^16^^,^^18^ the Monte Carlo modeling of light transport in multi-layered tissues (MCML) open-source code developed in the early nineties^19^ has been largely used as the standard reference. Due to this fact, the results of this code developed for a multi-layered medium can be considered as a numerical benchmark for photon migration through layered media. This special role played by MCML in biomedical applications is not observed for other MC codes.

One drawback of using verified MC codes to generate reference data is the limited accuracy that is achievable with a computer simulation. However, this drawback has not actually restricted the use of the MC method. In recent years, the facilitated access to open-source platforms of MC codes has made MC results easily available to a wider audience, and the practice of using verified MC codes has become the widest verification method used in biomedical applications.

It is also important to stress that in biomedical optics there is limited use of exact solutions of the radiative transfer equation (RTE) for the verification of MC codes. Indeed, only a few examples can be found for this kind of verification.^12^^,^^16^^,^^19^ This fact is related to the intrinsic complexity of both older and newly available solutions of the RTE,^20^^–^^23^ which are not in closed-form and require some numerical evaluation. The verification of MCML, and other MC codes, has been largely based on the RTE solutions tabulated by van de Hulst,^24^^,^^25^ related to a scattering slab, and on the RTE solutions for a semi-infinite medium tabulated by Giovanelli.^26^ The extensive use of these historical results from Giovanelli and Van de Hulst, even though affected by a limited accuracy, provides another clear indicator of the difficulties to use other benchmarks based on more complex newly available solutions of the RTE.^20^^–^^23^

The intent of this work is to propose a verification method purely based on the exact analytical solutions of the RTE. The selected benchmarks have the common characteristic to be extremely simple in terms of implementation so that the computational burden found with newly available RTE solutions^20^^–^^23^ is avoided. Therefore, their use is also open to the non-expert users of complex computational methods since their implementation is straightforward. The method is framed in two steps which focus on two different aspects of photon migration in scattering media: (a) propagation through homogeneous domains, where the statistical rules valid in an infinite medium for the extraction of photons’ trajectories are used^2^^,^^27^^,^^28^ and (b) propagation through regions with different optical properties, where the effects of boundaries must be accounted for.^19^^,^^27^^,^^28^ Accordingly to this vision any verification method of an MC code can be in principle divided into (a) verification in an infinite medium where the basic routines/algorithms to extract the photons’ trajectories can be tested by using very intrinsic analytical benchmarks of photons transport and (b) verification in finite media, or in media with a least one finite dimension, where the effects of boundaries can be tested by means of specific benchmarks.

Thus, as the first step, we propose to verify an MC code by using statistical formulas for the first and the second moments of the spatial coordinates where the different scattering orders occur in an infinite non-absorbing medium.^2^ For the second step of the verification method, we propose to use the invariant solutions for the radiance, I, the fluence rate, Φ, and mean total path length, ⟨L⟩, in bounded non-absorbing media subjected to Lambertian illumination.^3^^,^^29^^–^^31^ With this two-step procedure, all the main quantities involved in an MC simulation can be verified with a high level of accuracy. Finally, it can be noted that this procedure is suitable to be used during the development of an MC code, i.e., from its very beginning till the finalization of the code. This aspect is not secondary in our proposed method and has an important didactic value: instead of verifying a code at the end of its implementation, a verification “in progress” has the advantage to check the different algorithms and routines during their construction with the advantage that the effects of multiple bugs can be better detected.

It is worth noting that the proposed method largely extends the verification methods previously published in Refs. 2 and 3. The benchmarks used in Ref. 2 for isotropic scattering can now be applied for any scattering order. Compared to Ref. 3, the benchmarks used include fundamental radiometric quantities such as radiance and fluence rate. This latter extension is of fundamental interest because the radiance is at the origin of all the radiometric quantities used in radiative transfer, therefore a verification based on radiance is quite relevant. Moreover, the fluence rate represents a fundamental quantity in the latest generation of MC codes describing propagation in complex geometries,^6^^–^^14^^,^^16^^,^^17^ therefore, a verification method based on fluence rate is of the utmost importance.

In Sec. 2, the analytical benchmarks used in the proposed verification method are described. In the same section, the proposed verification method is also described. In Sec. 3, the results obtained with the two-step verification method are presented. In Sec. 4, the results described in Sec. 3 are briefly discussed together with the future perspectives for the application of this method.

## 2 Theory and Methods

This section describes the two kinds of benchmarks used in the verification procedure proposed in this work together with a brief description of the method.

### 2.1 Analytical Benchmarks for Light Propagation through an Infinite Medium: Statistical Moments of the Coordinates of the Scattering Events

In this section, the relationships existing between the statistical moments of the coordinates where scattering events occur and the optical properties are presented for the case of an infinite non-absorbing medium. It is at first considered the general case of rotationally symmetric scattering phase functions, i.e., scattering functions which can be represented as p(s^·s^′), with $\hat{s}$ direction of the incident radiation and s^′ direction of the scattered radiation (Sec. 2.1.1). Then, it is considered the special case of isotropic scattering (Appendix). The benchmarks presented in this section are a set of analytical solutions of immediate implementation. The proof of their validity can be found in the related cited literature (see below) and in the Appendix included in this work.

#### 2.1.1 Rotationally symmetric scattering phase functions

Let’s consider an infinite homogeneous non-absorbing medium, where at the origin of a Cartesian reference system we have a pencil light source in the z direction. The optical properties of the medium are described by the scattering coefficient, $\mu_{s}$, and the scattering function, p(θ), with θ the scattering angle.^27^^,^^28^ Although we consider a non-absorbing medium, it is important to note that the inclusion of absorption is straightforward by means of the microscopic Beer–Lambert law (mBLL).^28^ In what follows the average values of the first and second moments of the coordinates where the different scattering orders occur (up to the fourth-order) are reported.^2^ The different scattering orders will be denoted with a subscript giving the number of the scattering order. Thus, the mean values of $x_{1}$, $y_{1}$, and $z_{1}$ (first moments) where the first scattering event occurs are given by^2^

$$\langle x_{1}\rangle =\langle y_{1}\rangle =0,$$ (1)

$$\langle z_{1}\rangle =\frac{1}{\mu_{s}},$$ (2)

and the relative mean square values $\left\langle x_{1}^{2}\right\rangle$, $\left\langle y_{1}^{2}\right\rangle$, and $\left\langle z_{1}^{2}\right\rangle$ (second moments) are given by^2^

$$\langle x_{1}^{2}\rangle =\langle y_{1}^{2}\rangle =0,$$ (3)

$$\langle z_{1}^{2}\rangle =\frac{2}{\mu_{s}^{2}}.$$ (4)

From the above relations, the values of the mean square distance from the z axis, $\left\langle \rho_{1}^{2}\right\rangle$, and the mean square distance from the source, $\left\langle d_{1}^{2}\right\rangle$, are obtained as^2^

$$\langle \rho_{1}^{2}\rangle =\langle x_{1}^{2}+y_{1}^{2}\rangle =\langle x_{1}^{2}\rangle +\langle y_{1}^{2}\rangle =0,$$ (5)

$$\langle d_{1}^{2}\rangle =\langle x_{1}^{2}+y_{1}^{2}+z_{1}^{2}\rangle =\langle x_{1}^{2}\rangle +\langle y_{1}^{2}\rangle +\langle z_{1}^{2}\rangle =\frac{2}{\mu_{s}^{2}}.$$ (6)

For the second scattering order, we have^2^

$$\langle x_{2}\rangle =\langle y_{2}\rangle =0,$$ (7)

$$\langle z_{2}\rangle =\frac{1+g}{\mu_{s}},$$ (8)

$$\langle x_{2}^{2}\rangle =\langle y_{2}^{2}\rangle =\frac{1-g_{2}}{\mu_{s}^{2}},$$ (9)

$$\langle z_{2}^{2}\rangle =\frac{2(1+g+g_{2})}{\mu_{s}^{2}},$$ (10)

where g=⟨cos θ⟩ and $g_{2}=\langle \cos^{2}\;\theta \rangle$ are the first and second moments of the cosine of the scattering angle, respectively. The parameter g is usually denoted asymmetry factor of the phase scattering function.

From Eqs. (9) and (10) we have^2^

$$\langle \rho_{2}^{2}\rangle =\langle x_{2}^{2}+y_{2}^{2}\rangle =\langle x_{2}^{2}\rangle +\langle y_{2}^{2}\rangle =\frac{2(1-g_{2})}{\mu_{s}^{2}},$$ (11)

$$\langle d_{2}^{2}\rangle =\langle x_{2}^{2}+y_{2}^{2}+z_{2}^{2}\rangle =\langle x_{2}^{2}\rangle +\langle y_{2}^{2}\rangle +\langle z_{2}^{2}\rangle =\frac{2(2+g)}{\mu_{s}^{2}}.$$ (12)

The mean coordinates of the point at which the third-order of scattering occurs are^2^

$$\langle x_{3}\rangle =\langle y_{3}\rangle =0,$$ (13)

$$\langle z_{3}\rangle =\frac{1+g+g^{2}}{\mu_{s}},$$ (14)

and also

$$\langle x_{3}^{2}\rangle =\langle y_{3}^{2}\rangle =\frac{\frac{3}{2}+g-gg_{2}-\frac{3}{2}g_{2}^{2}}{\mu_{s}^{2}},$$ (15)

$$\langle z_{3}^{2}\rangle =\frac{3+2g+2g^{2}+2gg_{2}+3g_{2}^{2}}{\mu_{s}^{2}}.$$ (16)

Similarly to the previous scattering orders, we also have

$$\langle \rho_{3}^{2}\rangle =\langle x_{3}^{2}+y_{3}^{2}\rangle =\langle x_{3}^{2}\rangle +\langle y_{3}^{2}\rangle =\frac{3+2g-2gg_{2}-3g_{2}^{2}}{\mu_{s}^{2}},$$ (17)

$$\langle d_{3}^{2}\rangle =\langle x_{3}^{2}+y_{3}^{2}+z_{3}^{2}\rangle =\langle x_{3}^{2}\rangle +\langle y_{3}^{2}\rangle +\langle z_{3}^{2}\rangle =\frac{2(3+2g+g^{2})}{\mu_{s}^{2}}.$$ (18)

For the fourth-order, we have^2^

$$\langle x_{4}\rangle =\langle y_{4}\rangle =0,$$ (19)

$$\langle z_{4}\rangle =\frac{1+g+g^{2}+g^{3}}{\mu_{s}},$$ (20)

$$\langle x_{4}^{2}\rangle =\langle y_{4}^{2}\rangle =\frac{\frac{9}{4}+\frac{3}{2}g-\frac{3}{2}gg_{2}^{2}+g^{2}-g^{2}g_{2}-\frac{3}{4}g_{2}+\frac{3}{4}g_{2}^{2}-\frac{9}{4}g_{2}^{3}}{\mu_{s}^{2}},$$ (21)

$$\langle z_{4}^{2}\rangle =\frac{\frac{7}{2}+3g(1+g_{2}^{2})+2g^{2}(1+g_{2})+2g^{3}+\frac{3}{2}g_{2}-\frac{3}{2}g_{2}^{2}+\frac{9}{2}g_{2}^{3}}{\mu_{s}^{2}}.$$ (22)

$$\langle \rho_{4}^{2}\rangle =\langle x_{4}^{2}+y_{4}^{2}\rangle =\langle x_{4}^{2}\rangle +\langle y_{4}^{2}\rangle =\frac{\frac{9}{2}+3g-3gg_{2}^{2}+2g^{2}-2g^{2}g_{2}+-\frac{3}{2}g_{2}+\frac{3}{2}g_{2}^{2}-\frac{9}{2}g_{2}^{3}}{\mu_{s}^{2}},$$ (23)

$$\langle d_{4}^{2}\rangle =\langle x_{4}^{2}+y_{4}^{2}+z_{4}^{2}\rangle =\langle x_{4}^{2}\rangle +\langle y_{4}^{2}\rangle +\langle z_{4}^{2}\rangle =\frac{2(4+3g+2g^{2}+g^{3})}{\mu_{s}^{2}}.$$ (24)

The calculations can be in principle iterated for the higher scattering orders. Given the cylindrical symmetry, with respect to the z axis, we have that $\left\langle x_{k}\right\rangle$ and $\left\langle y_{k}\right\rangle$ are zero for any k. While for $\left\langle z_{k}\right\rangle$, the following relation is valid, which is given by^2^

$$\langle z_{k}\rangle =\frac{1-g^{k}}{\mu_{s}(1-g)}.$$ (25)

It is worth noting that the above equation for k→∞ returns the classical transport mean free path, $\frac{1}{\mu_{s}(1-g)}$.^28^ For the higher scattering orders it is also possible to extrapolate the following recurring relation for $\left\langle d_{k}^{2}\right\rangle$, which is given by^2^

$$\langle d_{k}^{2}\rangle =\langle x_{k}^{2}+y_{k}^{2}+z_{k}^{2}\rangle =2\frac{k-(k+1)g+g^{k+1}}{\mu_{s}^{2}{\left(1-g\right)}^{2}}.$$ (26)

A full proof of Eq. (26), derived from the RTE, was obtained by Liemert et al.^32^ for arbitrary rotationally symmetric scattering phase functions (e.g., the Henyey–Greenstein (HG) phase function).^28^ In Ref. 32, this expression is also generalized for an absorbing medium. The importance of this benchmark is that it can be used for any scattering order and is usually employed in biomedical optics for most of the scattering functions.

Statistical relationships can also be obtained for the optical path lengths of the propagated photons. The statistical moment of order m of the path length traveled at the k’th scattering event is given by^2^

$$\langle l_{k}^{m}\rangle =\frac{k(k+1)\cdots (k+m-1)}{\mu_{s}^{m}}.$$ (27)

#### 2.1.2 Isotropic scattering phase functions

For the case of isotropic scattering, it is possible to *extrapolate* the following recurring relations for the second moment for any scattering order k>0 as given by

$$\langle x_{k}^{2}\rangle =\langle y_{k}^{2}\rangle =\frac{2}{3\mu_{s}^{2}}(k-1),$$ (28)

$$\langle z_{k}^{2}\rangle =\frac{2}{3\mu_{s}^{2}}(k+2),$$ (29)

$$\langle \rho_{k}^{2}\rangle =\langle x_{k}^{2}+y_{k}^{2}\rangle =\langle x_{k}^{2}\rangle +\langle y_{k}^{2}\rangle =\frac{4}{3\mu_{s}^{2}}(k-1),$$ (30)

$$\langle d_{k}^{2}\rangle =\langle x_{k}^{2}+y_{k}^{2}+z_{k}^{2}\rangle =\langle x_{k}^{2}\rangle +\langle y_{k}^{2}\rangle +\langle z_{k}^{2}\rangle =\frac{2k}{\mu_{s}^{2}}.$$ (31)

The proof of the validity of the above equations is treated in the Appendix where exact analytical equations for the moments of the coordinates where scattering events occur are obtained. We notice that, compared to the previous benchmarks (Sec. 2.1.1), the calculated moments for isotropic scattering do not depend on g and $g_{2}$.

The presented benchmarks provide an overview of the “cloud” of photons migrating in an infinite medium. The first moments yield the coordinates of its barycenter, while the second central moments represent its width along the three axes. They are strictly related to the scattering function and the statistical law for scattering interactions. The features of the scattering function that affect the statistical moments are: g=⟨cos θ⟩ and $g_{2}=\langle \cos^{2}\;\theta \rangle$. Thus, these benchmarks are suitable to verify if an MC code correctly extracts the photons’ trajectories and consequently the scattering function is correctly implemented inside the code.

### 2.2 Analytical Benchmarks for Boundary Effects: Invariant Solutions for Radiance, Fluence Rate, and Total Mean Path Length

Photon migration in a finite medium is also characterized by boundary effects, i.e., reflection, refraction, and intersection. Besides the boundary with the outer medium, internal boundaries are also used to enclose regions with different optical properties. The correct evaluation of boundary effects is another important task of an MC code. To verify the reliability of an MC code to simulate boundary effects, we propose to use a set of invariant exact solutions of the RTE for the radiance, fluence rate, and for the partial and total path lengths that are obtained when a *non-absorbing* medium is subjected to a Lambertian illumination.^29^^,^^30^^,^^33^^,^^34^ These solutions depend on the distribution of the refractive index inside the medium relative to the refractive index of the outer medium.

Let’s consider a non-absorbing inhomogeneous medium of volume V (with at least one finite dimension) without internal sources, delimited by a smooth convex surface Σ. The medium is composed of a number N of discrete sub-volumes $V_{j}$ of refractive index $n_{j}$ and with no restrictions on the scattering properties inside each $V_{j}$. The surfaces enclosing each sub-volume are assumed to be smooth so that Snell’s and Fresnel’s law can be applied. The refractive index of the external medium is denoted with $n_{e}$ and the surface Σ is illuminated by a continuous wave Lambertian radiation of intensity $I_{0}(W\;m^{-2}\;{\mathrm{sr}}^{-1})$, i.e., the source term is thus can be expressed in terms of the distribution of radiance on the external surface Σ, which is given by

IeSource(r→Σ,s^e)=I0[W m−2 sr−1]∀  r→Σ∈Σand∀  s^e  inwardly directed to  Σ. (32)

In this case, the solution for the radiance inside $V_{j}$ is^29^

Ij(r→,s^)=(njne)2I0 [W m−2 sr−1]∀  s^and∀  r→∈Vj, (33)

where $I_{j}(\vec{r},\hat{s})$ is a function of the refractive indices $n_{j}$, internal to $V_{j}$, and $n_{e}$, external to V; however, we notice that this solution does not depend on the refractive index of the remaining sub-volumes. Also, in Eq. (33) $\hat{s}$ denotes the direction vector, while $\vec{r}$ denotes the position vector internal to the medium. Consequently, the solution for the fluence inside $V_{j}$ is expressed as^29^

Φj(r→)=∫4πIj(r→,s^)ds^=4π(njne)2I0,  [W m−2],∀  r→∈Vj. (34)

Finally, the average internal path length in each sub-volume $V_{j}$ results in^29^

$$\langle L_{j}\rangle =4{\left(\frac{n_{j}}{n_{e}}\right)}^{2}\frac{V_{j}}{\Sigma }.$$ (35)

The average total path length ⟨L⟩ inside the total volume V can also be evaluated by simply summing up all the contributions of the average internal path lengths $\left\langle L_{j}\right\rangle$ of Eq. (35), i.e.,

$$\langle L\rangle =\overset{N}{\underset{j=1}{\sum }}\langle L_{j}\rangle =4\overset{N}{\underset{j=1}{\sum }}{\left(\frac{n_{j}}{n_{e}}\right)}^{2}\frac{V_{j}}{\Sigma }.$$ (36)

From Eqs. (33) and (34), we clearly see that the solutions for the internal radiance and the fluence rate are invariant both with respect to the geometry of the medium (provided that the external surface is convex and smooth) and with respect to the distribution of the scattering properties (scattering coefficient, scattering function, and homogeneity). Similarly, the expressions for the average internal path lengths $\left\langle L_{j}\right\rangle$ and the total path length ⟨L⟩ are invariant with respect to the scattering properties of the medium, however, they depend (as it is expected) on the volumes of the sub-regions and the whole external surface Σ. One exception is the case of $\mu_{sj}(\vec{r})=0$ whenever the geometry (e.g., sphere or slab) of the medium allows for a regime of trapped trajectories.^29^ These properties imply that these benchmarks can be used for any scattering coefficient of the medium except, with $\mu_{sj}(\vec{r})=0$, for all those geometries where trapped trajectories can be established.

In general, for a non-scattering medium [$\mu_{sj}(\vec{r})=0$] with $n_{j}\leq n_{e}$, the above solutions are still valid. While, with $\mu_{sj}(\vec{r})=0$, for geometries like a sphere or slab with $n_{j}>n_{e}$,^29^^,^^35^ where photons can be trapped inside the volume, the same RTE solutions found for scattering media cannot be used. For example, the RTE solution for a layered non-scattering slab (which will be used in the results section) should account for the regime of trapped photons. Accordingly, the RTE solution for the internal radiance inside a layered non-scattering slab is^29^

Ij(r→,s^)=(njne)2I0∀  r→∈Vj∀  s^||s^·q^|≥cos θj MaxIj(r→,s^)=0∀  r→∈Vj∀  s^||s^·q^|<cos θj Max, (37)

where $I_{0}$ is the radiance on the external surface Σ, $\hat{q}$ is the unit vector perpendicular to the slab inwardly directed, $\theta_{j\;\mathrm{Max}}$ is the maximum entrance angle in the medium for having radiation in the j’th layer. Consequently, the solution for the fluence rate in a layered slab is

$$\Phi_{j}(\overset{\to }{r})=4\pi {\left(\frac{n_{j}}{n_{e}}\right)}^{2}I_{0}[1-\cos(\theta_{j\;\mathrm{Max}})],\;[W\;m^{-2}]\;\forall \;\overset{\to }{r}\in V_{j}.$$ (38)

Finally, the average internal path length $\left\langle L_{j}\right\rangle$ spent in the j’th layer of the slab is

$$\langle L_{j}\rangle =2s_{j}{\left(\frac{n_{j}}{n_{e}}\right)}^{2}[1-\cos(\theta_{j\;\mathrm{Max}})],$$ (39)

where $s_{j}$ is the thickness of the j’th layer of the slab. For some guidelines to calculate the angle $\theta_{j\;\mathrm{Max}}$, we refer to the work of Martelli et al.^29^ It may be worth noting that for $n_{j}>n_{e}$ in the above solutions we have a discontinuity of the radiance between the case $\mu_{s}=0$ [Eq. (37)] and the case $\mu_{s}\neq 0$ [Eq. (33)].^29^ When $n_{j}>n_{e}$, a discontinuity is also observed for the fluence rate, $\Phi_{j}$ and the partial path length $\left\langle L_{j}\right\rangle$ as can be similarly noted by the above equations. ^29^^,^^35^ Moreover, when $n_{j}>n_{e}$ and $\mu_{s}=0$, from Eq. (37) the discontinuity of the radiance can be observed for the angle $\theta_{j\;\mathrm{Max}}$. At such value of the angle the radiance $I_{j}$ switches from the value $(\frac{n_{j}}{n_{e}}{)}^{2}I_{0}$ to zero.

The presented benchmarks provide an overview of invariant solutions of the RTE in non-absorbing media that are sensitive to the effects of boundary conditions between the different regions of the medium and also between the medium and the external region. It must be noted that the independence of the presented solutions from the scattering properties of the medium implies that they cannot be used to test the correctness of the phase function implemented in the code. However, this is done with the previous benchmark of Sec. 2.1. Combined together, the benchmarks of Secs. 2.1 and 2.2 cover the main characteristics of photon migration according to the RTE.

### 2.3 Proposed Method

The verification method here proposed is divided into two steps and exploits the following strategy: (1) all the routines of the code involved in the extraction of the trajectories, based also on the scattering function are verified by a direct comparison of MC results in an infinite medium with the benchmarks as in Sec. 2.1 and (2) the routines of the MC code related to the interactions of photons’ trajectories with boundaries are verified by a direct comparison of the MC results in a slab geometry with the invariant solutions for radiance, fluence rate, and mean path length as in Sec. 2.2.

Concisely, the first step is devised to test the phase function implemented in the MC code, the second one to test the intersection of photon’s trajectories with boundaries, including the correct implementation of the reflection and refraction laws. The second step includes the boundary with the external medium and those between regions of the medium with different optical properties. We notice that the correct calculation of the intersections with boundaries is at the core of partial path length estimation, which is one fundamental task of an MC code. In fact, the absence of boundaries in the first step is ideal for testing the phase function. In contrast, the invariant solutions of RTE used in the second step (which do not depend on the features of the phase function) are ideal for testing the intersection with boundaries.

We believe that the two-step verification increases the sensitivity to detect errors in an MC code.

## 3 Results

In this section, the results obtained using the proposed method in the two steps of the verification are presented. The results obtained in the first step of the verification are described in Sec. 3.1. While the results obtained in the second step of the verification are described in Sec. 3.2.

### 3.1 First step of the Verification (Sec. 2.1)

In this section, the moments of the coordinates of the scattering points calculated with MC simulations have been compared with the analytical benchmarks of Sec. 2.1. All the comparisons are carried out in an infinite non-absorbing medium with a unitary scattering coefficient $\mu_{s}=1\;{\mathrm{mm}}^{-1}$ where a pencil beam source is injected at the origin of a Cartesian reference system along the z direction. Three scattering functions have been selected: the HG model with g=0 and 0.9,^28^ and a Rayleigh scattering function (g=0).^28^ This choice is motivated on this ground: for these scattering functions the moments g and $g_{2}$ are known exactly without resorting to numerical evaluations. This is not true for phase scattering functions derived from Mie theory.^28^

The MC code here subjected to the verification procedure is a program used for simulating light propagation in tissue optics. The core of the program, developed during the period 1980 to 1999,^28^^,^^36^^–^^39^ generates a large number of photons’ trajectories for different scattering coefficients and refractive indices. In Tables 1–3 the relative errors (ratio between standard error and average value) of the MC results obtained for the moments of Sec. 2.1 for the first four scattering orders (k∈{1,…,4}) are shown for three values of the number of simulated trajectories $N\in \{10^{6},10^{8},10^{10}\}$. We notice that the relative error decreases by one order of magnitude as the value of N increases by two orders of magnitude. This behavior expresses exactly the expected distributions for the calculated quantities and highlights the fact that the precision of the calculation is only limited by the numerical accuracy of the processor and by the number of simulated trajectories, as is expected from the statistical theory. In these tables, the data for $\left\langle x_{1}\right\rangle$, $\left\langle y_{1}\right\rangle$, $\left\langle x_{1}^{2}\right\rangle$, $\left\langle y_{1}^{2}\right\rangle$, and $\left\langle \rho_{1}^{2}\right\rangle$ have not been reported since their values are null and the relative uncertainty cannot be calculated.

**Table 1:** Relative uncertainty, $\frac{\sigma_{{\left\langle \ldots \right\rangle }_{\mathrm{MC}}}}{{\left\langle \ldots \right\rangle }_{\mathrm{MC}}}$, of the statistical moments of the coordinates of scattering events calculated with MC simulations for a non-absorbing infinite medium with scattering coefficient $\mu_{s}=1\;{\mathrm{mm}}^{-1}$, isotropic scattering function obtained with the HG model (g=0, $g_{2}=1/3$), and three values N of generated trajectories.

$\frac{\sigma_{{\left\langle \ldots \right\rangle }_{\mathrm{MC}}}}{{\left\langle \ldots \right\rangle }_{\mathrm{MC}}}$	$N=10^{6}$				$N=10^{8}$				$N=10^{10}$			
k	1	2	3	4	1	2	3	4	1	2	3	4
$\left\langle z_{k}\right\rangle$	$1.000\cdot 10^{-3}$	$1.291\cdot 10^{-3}$	$1.529\cdot 10^{-3}$	$1.732\cdot 10^{-3}$	$1.000\cdot 10^{-4}$	$1.291\cdot 10^{-4}$	$1.528\cdot 10^{-4}$	$1.732\cdot 10^{-4}$	$1.000\cdot 10^{-5}$	$1.291\cdot 10^{-5}$	$1.528\cdot 10^{-5}$	$1.732\cdot 10^{-5}$
$\left\langle x_{k}^{2}\right\rangle$		$3.125\cdot 10^{-3}$	$2.436\cdot 10^{-3}$	$2.152\cdot 10^{-3}$		$3.133\cdot 10^{-4}$	$2.429\cdot 10^{-4}$	$2.145\cdot 10^{-4}$		$3.130\cdot 10^{-5}$	$2.429\cdot 10^{-5}$	$2.145\cdot 10^{-5}$
$\left\langle y_{k}^{2}\right\rangle$		$3.134\cdot 10^{-3}$	$2.428\cdot 10^{-3}$	$2.141\cdot 10^{-3}$		$3.132\cdot 10^{-4}$	$2.429\cdot 10^{-4}$	$2.145\cdot 10^{-4}$		$3.131\cdot 10^{-5}$	$2.429\cdot 10^{-5}$	$2.145\cdot 10^{-5}$
$\left\langle z_{k}^{2}\right\rangle$	$2.229\cdot 10^{-3}$	$2.042\cdot 10^{-3}$	$1.927\cdot 10^{-3}$	$1.845\cdot 10^{-3}$	$2.236\cdot 10^{-4}$	$2.043\cdot 10^{-4}$	$1.925\cdot 10^{-4}$	$1.845\cdot 10^{-4}$	$2.236\cdot 10^{-5}$	$2.043\cdot 10^{-5}$	$1.925\cdot 10^{-5}$	$1.844\cdot 10^{-5}$
$\left\langle \rho_{k}^{2}\right\rangle$		$2.486\cdot 10^{-3}$	$1.899\cdot 10^{-3}$	$1.655\cdot 10^{-3}$		$2.492\cdot 10^{-4}$	$1.898\cdot 10^{-4}$	$1.653\cdot 10^{-4}$		$2.490\cdot 10^{-5}$	$1.897\cdot 10^{-5}$	$1.653\cdot 10^{-5}$
$\left\langle d_{k}^{2}\right\rangle$	$2.229\cdot 10^{-3}$	$1.682\cdot 10^{-3}$	$1.455\cdot 10^{-3}$	$1.323\cdot 10^{-3}$	$2.236\cdot 10^{-4}$	$.684\cdot 10^{-4}$	$1.453\cdot 10^{-4}$	$1.323\cdot 10^{-4}$	$2.236\cdot 10^{-5}$	$1.683\cdot 10^{-5}$	$1.453\cdot 10^{-5}$	$1.323\cdot 10^{-5}$
$\left\langle l_{k}\right\rangle$	$1.000\cdot 10^{-3}$	$7.077\cdot 10^{-4}$	$5.776\cdot 10^{-4}$	$4.999\cdot 10^{-4}$	$1.000\cdot 10^{-4}$	$7.071\cdot 10^{-5}$	$5.773\cdot 10^{-5}$	$5.001\cdot 10^{-5}$	$1.000\cdot 10^{-5}$	$7.071\cdot 10^{-6}$	$5.774\cdot 10^{-6}$	$5.000\cdot 10^{-6}$

**Table 2:** Relative uncertainty, $\frac{\sigma_{{\left\langle \ldots \right\rangle }_{\mathrm{MC}}}}{{\left\langle \ldots \right\rangle }_{\mathrm{MC}}}$, of the statistical moments of the coordinates of scattering events calculated with MC simulations for a non-absorbing infinite medium with scattering coefficient $\mu_{s}=1\;{\mathrm{mm}}^{-1}$, an anisotropic scattering function obtained with the HG model with g=0.9 ($g_{2}=2.62/3$), and three values N of generated trajectories.

$\frac{\sigma_{{\left\langle \ldots \right\rangle }_{\mathrm{MC}}}}{{\left\langle \ldots \right\rangle }_{\mathrm{MC}}}$	$N=10^{6}$				$N=10^{8}$				$N=10^{10}$			
k	1	2	3	4	1	2	3	4	1	2	3	4
$\left\langle z_{k}\right\rangle$	$1.000\cdot 10^{-3}$	$7.330\cdot 10^{-4}$	$6.331\cdot 10^{-4}$	$5.884\cdot 10^{-4}$	$1\cdot 10^{-4}$	$7.324\cdot 10^{-6}$	$6.322\cdot 10^{-6}$	$5.880\cdot 10^{-5}$	$1.000\cdot 10^{-5}$	$7.324\cdot 10^{-6}$	$6.323\cdot 10^{-6}$	$5.880\cdot 10^{-6}$
$\left\langle x_{k}^{2}\right\rangle$		$6.019\cdot 10^{-3}$	$3.795\cdot 10^{-3}$	$2.957\cdot 10^{-3}$		$6.009\cdot 10^{-4}$	$3.847\cdot 10^{-4}$	$2.986\cdot 10^{-4}$		$6.009\cdot 10^{-5}$	$3.846\cdot 10^{-5}$	$2.986\cdot 10^{-5}$
$\left\langle y_{k}^{2}\right\rangle$		$5.931\cdot 10^{-3}$	$3.801\cdot 10^{-3}$	$2.967\cdot 10^{-3}$		$5.993\cdot 10^{-4}$	$3.845\cdot 10^{-4}$	$2.983\cdot 10^{-4}$		$6.007\cdot 10^{-5}$	$3.845\cdot 10^{-5}$	$2.986\cdot 10^{-5}$
$\left\langle z_{k}^{2}\right\rangle$	$2.229\cdot 10^{-3}$	$1.564\cdot 10^{-3}$	$1.294\cdot 10^{-3}$	$1.148\cdot 10^{-3}$	$2.236\cdot 10^{-4}$	$1.564\cdot 10^{-4}$	$1.293\cdot 10^{-4}$	$1.148\cdot 10^{-4}$	$2.236\cdot 10^{-5}$	$1.564\cdot 10^{-5}$	$1.293\cdot 10^{-5}$	$1.148\cdot 10^{-5}$
$\left\langle \rho_{k}^{2}\right\rangle$		$4.838\cdot 10^{-3}$	$3.054\cdot 10^{-3}$	$2.352\cdot 10^{-3}$		$4.865\cdot 10^{-4}$	$3.086\cdot 10^{-4}$	$2.367\cdot 10^{-4}$		$4.871\cdot 10^{-5}$	$3.086\cdot 10^{-5}$	$2.369\cdot 10^{-5}$
$\left\langle d_{k}^{2}\right\rangle$	$2.229\cdot 10^{-3}$	$1.528\cdot 10^{-3}$	$1.225\cdot 10^{-3}$	$1.048\cdot 10^{-3}$	$2.236\cdot 10^{-4}$	$1.538\cdot 10^{-4}$	$1.244\cdot 10^{-4}$	$1.076\cdot 10^{-4}$	$2.236\cdot 10^{-5}$	$1.538\cdot 10^{-5}$	$1.244\cdot 10^{-5}$	$1.076\cdot 10^{-5}$
$\left\langle l_{k}\right\rangle$	$1.000\cdot 10^{-3}$	$7.077\cdot 10^{-3}$	$5.776\cdot 10^{-4}$	$4.999\cdot 10^{-4}$	$1.000\cdot 10^{-4}$	$7.071\cdot 10^{-5}$	$5.773\cdot 10^{-5}$	$5.001\cdot 10^{-5}$	$1.000\cdot 10^{-5}$	$7.071\cdot 10^{-6}$	$5.774\cdot 10^{-6}$	$5.000\cdot 10^{-6}$
$\left\langle l_{k}^{2}\right\rangle$	$2.229\cdot 10^{-3}$	$1.528\cdot 10^{-3}$	$1.225\cdot 10^{-3}$	$1.048\cdot 10^{-3}$	$2.236\cdot 10^{-4}$	$1.528\cdot 10^{-4}$	$1.225\cdot 10^{-4}$	$1.049\cdot 10^{-4}$	$2.236\cdot 10^{-5}$	$1.528\cdot 10^{-5}$	$1.225\cdot 10^{-5}$	$1.049\cdot 10^{-5}$

**Table 3:** Relative uncertainty, $\frac{\sigma_{\langle \ldots \rangle_{\mathrm{MC}}}}{\langle \ldots \rangle_{\mathrm{MC}}}$, of the statistical moments of the coordinates of scattering events calculated with MC simulations for a non-absorbing infinite medium with scattering coefficient $\mu_{s}=1\;{\mathrm{mm}}^{-1}$, a Rayleigh scattering function (g=0, $g_{2}=0.4$), and three values N of generated trajectories.

$\frac{\sigma_{\langle \ldots \rangle_{\mathrm{MC}}}}{\langle \ldots \rangle_{\mathrm{MC}}}$	$N=10^{6}$				$N=10^{8}$				$N=10^{10}$			
k	1	2	3	4	1	2	3	4	1	2	3	4
$\left\langle z_{k}\right\rangle$	$1.000\cdot 10^{-3}$	$1.339\cdot 10^{-3}$	$1.573\cdot 10^{-3}$	$1.770\cdot 10^{-3}$	$1.000\cdot 10^{-4}$	$1.342\cdot 10^{-4}$	$1.575\cdot 10^{-4}$	$1.774\cdot 10^{-4}$	$1.000\cdot 10^{-5}$	$1.342\cdot 10^{-5}$	$1.575\cdot 10^{-5}$	$1.774\cdot 10^{-5}$
$\left\langle x_{k}^{2}\right\rangle$		$3.222\cdot 10^{-3}$	$2.475\cdot 10^{-3}$	$2.185\cdot 10^{-3}$		$3.232\cdot 10^{-4}$	$2.490\cdot 10^{-4}$	$2.190\cdot 10^{-4}$		$3.229\cdot 10^{-5}$	$2.488\cdot 10^{-5}$	$2.189\cdot 10^{-5}$
$\left\langle y_{k}^{2}\right\rangle$		$3.233\cdot 10^{-3}$	$2.493\cdot 10^{-3}$	$2.177\cdot 10^{-3}$		$3.231\cdot 10^{-4}$	$2.488\cdot 10^{-4}$	$2.189\cdot 10^{-4}$		$3.229\cdot 10^{-5}$	$2.488\cdot 10^{-5}$	$2.188\cdot 10^{-5}$
$\left\langle z_{k}^{2}\right\rangle$	$2.229\cdot 10^{-3}$	$2.016\cdot 10^{-3}$	$1.912\cdot 10^{-3}$	$1.837\cdot 10^{-3}$	$2.236\cdot 10^{-4}$	$2.018\cdot 10^{-4}$	$1.912\cdot 10^{-4}$	$1.839\cdot 10^{-4}$	$2.236\cdot 10^{-5}$	$2.018\cdot 10^{-5}$	$1.912\cdot 10^{-5}$	$1.839\cdot 10^{-5}$
$\left\langle \rho_{k}^{2}\right\rangle$		$2.567\cdot 10^{-3}$	$1.942\cdot 10^{-3}$	$1.686\cdot 10^{-3}$		$2.575\cdot 10^{-4}$	$1.948\cdot 10^{-4}$	$1.691\cdot 10^{-4}$		$2.573\cdot 10^{-5}$	$1.948\cdot 10^{-5}$	$1.691\cdot 10^{-5}$
$\left\langle d_{k}^{2}\right\rangle$	$2.229\cdot 10^{-3}$	$1.702\cdot 10^{-3}$	$1.472\cdot 10^{-3}$	$1.339\cdot 10^{-3}$	$2.236\cdot 10^{-4}$	$1.703\cdot 10^{-4}$	$1.474\cdot 10^{-4}$	$1.343\cdot 10^{-4}$	$2.236\cdot 10^{-5}$	$1.703\cdot 10^{-5}$	$1.474\cdot 10^{-5}$	$1.343\cdot 10^{-5}$
$\left\langle l_{k}\right\rangle$	$1.000\cdot 10^{-3}$	$7.077\cdot 10^{-3}$	$5.776\cdot 10^{-4}$	$4.999\cdot 10^{-4}$	$1.000\cdot 10^{-4}$	$7.071\cdot 10^{-5}$	$5.773\cdot 10^{-5}$	$5.001\cdot 10^{-5}$	$1.000\cdot 10^{-5}$	$7.071\cdot 10^{-6}$	$5.774\cdot 10^{-6}$	$5.000\cdot 10^{-6}$
$\left\langle l_{k}^{2}\right\rangle$	$2.229\cdot 10^{-3}$	$1.528\cdot 10^{-3}$	$1.225\cdot 10^{-3}$	$1.048\cdot 10^{-3}$	$2.236\cdot 10^{-4}$	$1.528\cdot 10^{-4}$	$1.225\cdot 10^{-4}$	$1.049\cdot 10^{-4}$	$2.236\cdot 10^{-5}$	$1.528\cdot 10^{-5}$	$1.225\cdot 10^{-5}$	$1.049\cdot 10^{-5}$

To have clear information on the accuracy of the simulated results, in Tables 4–6 the deviation of the MC results from the benchmarks of Sec. 2.1 is shown. The deviation is normalized to the standard error of the MC results (this defines the t random variable to be used for the one-sample t-test). We found that all the MC results (with only one exception) were consistent with the RTE values within two standard errors. The t-tests for all the cases reported in Tables 4–6 at a 5% level of significance show that the null hypothesis is not rejected. These results testify to the consistency of the extracted trajectories with the statistical rules of propagation of photon transport. The expected values from the RTE are shown in Table 7. The MC code is also able to describe the small differences between the moments for pure isotropic scattering (HG, g=0) and Rayleigh scattering as can be noted in the reported tables.

**Table 4:** Deviation of the MC results from the theory normalized to the MC standard error, $\frac{{\left\langle \ldots \right\rangle }_{\mathrm{MC}}-{\left\langle \ldots \right\rangle }_{\mathrm{RTE}}}{\sigma_{{\left\langle \ldots \right\rangle }_{\mathrm{MC}}}}$, for the statistical moments of the coordinates of scattering events in a non-absorbing infinite medium with scattering coefficient $\mu_{s}=1\;{\mathrm{mm}}^{-1}$, isotropic scattering function obtained with the HG model (g=0, $g_{2}=1/3$), and three values N of generated trajectories.

$\frac{{\left\langle \ldots \right\rangle }_{\mathrm{MC}}-{\left\langle \ldots \right\rangle }_{\mathrm{RTE}}}{\sigma_{{\left\langle \ldots \right\rangle }_{\mathrm{MC}}}}$	$N=10^{6}$				$N=10^{8}$				$N=10^{10}$			
k	1	2	3	4	1	2	3	4	1	2	3	4
$\left\langle x_{k}\right\rangle$		−0.865	−0.483	0.271		−0.837	−0.480	−0.491		0.996	0.838	0.467
$\left\langle y_{k}\right\rangle$		0.461	0.052	0.532		−0.257	−0.096	0.798		1.046	−0.089	−0.198
$\left\langle z_{k}\right\rangle$	0.848	−0.162	−0.380	0.023	−0.817	−0.261	0.275	1.022	1.141	1.687	1.734	1.459
$\left\langle x_{k}^{2}\right\rangle$		−1.050	−0.051	−0.362		−0.817	1.244	0.118		−0.746	−0.328	−0.193
$\left\langle y_{k}^{2}\right\rangle$		−1.165	−0.642	−0.710		−0.023	−0.628	−0.244		0.561	0.538	0.093
$\left\langle z_{k}^{2}\right\rangle$	0.981	0.022	0.081	0.190	−0.878	−0.430	0.410	0.235	1.227	1.093	1.769	1.312
$\left\langle \rho_{k}^{2}\right\rangle$		−1.394	−0.378	−0.692		−0.528	0.394	−0.082		−0.116	0.134	−0.065
$\left\langle d_{k}^{2}\right\rangle$	0.982	−0.667	−0.159	−0.300	−0.878	−0.608	0.531	0.113	1.227	0.827	1.380	0.874
$\left\langle l_{k}\right\rangle$	0.848	−0.933	−0.400	−0.030	−0.817	−1.470	−0.191	−0.384	1.141	0.602	0.053	−0.012
$\left\langle l_{k}^{2}\right\rangle$	0.982	−0.525	−0.169	−0.085	−0.878	−1.410	−0.258	0.043	1.227	0.580	0.219	0.048

**Table 5:** Deviation of the MC results from the theory normalized to the MC standard error, $\frac{\langle \ldots \rangle_{\mathrm{MC}}-\langle \ldots \rangle_{\mathrm{RTE}}}{\sigma_{\langle \ldots \rangle_{\mathrm{MC}}}}$, for the statistical moments of the coordinates of scattering events in a non-absorbing infinite medium with scattering coefficient $\mu_{s}=1\;{\mathrm{mm}}^{-1}$, an anisotropic scattering function obtained with the HG model with g=0.9 ($g_{2}=2.62/3$), and three values N of generated trajectories.

$\frac{\langle \ldots \rangle_{\mathrm{MC}}-\langle \ldots \rangle_{\mathrm{RTE}}}{\sigma_{\langle \ldots \rangle_{\mathrm{MC}}}}$	$N=10^{6}$				$N=10^{8}$				$N=10^{10}$			
k	1	2	3	4	1	2	3	4	1	2	3	4
$\left\langle x_{k}\right\rangle$		−0.438	0.709	0.635		0.632	1.750	1.140		0.184	0.401	0.825
$\left\langle y_{k}\right\rangle$		0.714	−0.066	−0.450		−1.130	−1.869	−1.432		0.218	0.479	0.188
$\left\langle z_{k}\right\rangle$	0.848	−1.037	−0.854	−0.450	−0.817	−1.293	0.188	0.103	1.141	0.543	0.175	0.310
$\left\langle x_{k}^{2}\right\rangle$		0.493	0.158	0.527		−0.965	−1.323	−1.026		−1.717	−1.467	−0.280
$\left\langle y_{k}^{2}\right\rangle$		−0.021	−0.072	−0.240		−0.632	−0.189	−0.029		−0.807	−0.836	−0.932
$\left\langle z_{k}^{2}\right\rangle$	0.982	−0.615	−0.275	−0.162	−0.878	−1.289	−0.050	0.071	1.227	0.693	0.703	0.611
$\left\langle \rho_{k}^{2}\right\rangle$		0.294	0.0538	0.180		−0.985	−0.942	−0.665		−1.556	−1.435	−0.764
$\left\langle d_{k}^{2}\right\rangle$	0.982	−0.558	−0.251	−0.104	−0.878	−1.390	−0.244	−0.110	1.227	0.459	0.371	0.370
$\left\langle l_{k}\right\rangle$	0.848	−0.933	−0.400	−0.030	−0.817	−1.470	−0.191	−0.384	1.141	0.602	0.053	−0.012
$\left\langle l_{k}^{2}\right\rangle$	0.982	−0.526	−0.169	−0.085	−0.878	−1.410	−0.258	0.043	1.227	0.580	0.219	0.048

**Table 6:** Deviation of the MC results from the theory normalized to the MC standard error, $\frac{\langle \ldots \rangle_{\mathrm{MC}}-\langle \ldots \rangle_{\mathrm{RTE}}}{\sigma_{\langle \ldots \rangle_{\mathrm{MC}}}}$, for the statistical moments of the coordinates of scattering events in a non-absorbing infinite medium with scattering coefficient $\mu_{s}=1\;{\mathrm{mm}}^{-1}$, a Rayleigh scattering function (g=0, $g_{2}=0.4$), and three values N of generated trajectories.

$\frac{\langle \ldots \rangle_{\mathrm{MC}}-\langle \ldots \rangle_{\mathrm{RTE}}}{\sigma_{\langle \ldots \rangle_{\mathrm{MC}}}}$	$N=10^{6}$				$N=10^{8}$				$N=10^{10}$			
k	1	2	3	4	1	2	3	4	1	2	3	4
$\left\langle x_{k}\right\rangle$		−0.845	0.623	−0.092		−0.848	−0.964	−0.294		1.108	0.198	0.393
$\left\langle y_{k}\right\rangle$		0.044	−0.035	0.172		−0.235	0.072	−0.069		1.096	0.447	−0.308
$\left\langle z_{k}\right\rangle$	0.848	1.451	0.996	1.122	−0.817	−0.943	−0.382	−0.397	1.141	−0.031	0.268	0.555
$\left\langle x_{k}^{2}\right\rangle$		−1.044	−1.295	−0.723		−0.709	0.542	−0.556		−0.660	−1.455	−0.701
$\left\langle y_{k}^{2}\right\rangle$		−1.158	−0.0546	0.962		0.002	0.063	−0.142		0.486	−1.767	−1.660
$\left\langle z_{k}^{2}\right\rangle$	0.982	0.554	0.769	0.308	−0.878	−1.691	−0.747	−0.697	1.227	0.446	0.993	0.960
$\left\langle \rho_{k}^{2}\right\rangle$		−1.384	−1.170	−1.090		−0.444	0.386	−0.452		−0.109	−2.058	−1.527
$\left\langle d_{k}^{2}\right\rangle$	0.982	−0.164	−0.067	−0.441	−0.878	−1.604	−0.347	−0.769	1.227	0.320	−0.395	−0.245
$\left\langle l_{k}\right\rangle$	0.848	−0.933	−0.400	−0.030	−0.817	−1.470	−0.191	−0.384	1.141	0.602	0.053	−0.012
$\left\langle l_{k}^{2}\right\rangle$	0.982	−0.526	−0.169	−0.086	−0.878	−1.410	−0.258	0.043	1.227	0.580	0.219	0.048

**Table 7:** Expected values from the RTE theory of the statistical moments of the coordinates of scattering events for a non-absorbing infinite medium with scattering coefficient $\mu_{s}=1\;{\mathrm{mm}}^{-1}$ and three scattering functions: HG (g=0), HG (g=0.9), and Rayleigh.

RTE	HG (g=0)				HG (g=0.9)				Rayleigh			
k	1	2	3	4	1	2	3	4	1	2	3	4
$\left\langle x_{k}\right\rangle$ (mm)	0.	0.	0.	0.	0.	0.	0.	0.	0.	0.	0.	0.
$\left\langle y_{k}\right\rangle$ (mm)	0.	0.	0.	0.	0.	0.	0.	0.	0.	0.	0.	0.
$\left\langle z_{k}\right\rangle$ (mm)	1.	1.	1.	1.	1.	1.9	2.71	3.349	1.	1.	1.	1.
$\left\langle x_{k}^{2}\right\rangle$ (${\mathrm{mm}}^{2}$)	0.	$0.\overset{¯}{6}$	$1.\bar{3}$	2.	0.	$0.12\bar{6}$	0.469933	1.091246	0.	0.6	1.26	1.926
$\left\langle y_{k}^{2}\right\rangle$ (${\mathrm{mm}}^{2}$)	0.	$0.\overset{¯}{6}$	$1.\bar{3}$	2.	0.	$0.12\bar{6}$	0.469933	1.091246	0.	0.6	1.26	1.926
$\left\langle z_{k}^{2}\right\rangle$ (${\mathrm{mm}}^{2}$)	2.	$2.\bar{6}$	$3.\bar{3}$	4.	2.	$5.54\bar{6}$	10.28013	15.91551	2.	2.8	3.48	4.148
$\left\langle \rho_{k}^{2}\right\rangle$ (${\mathrm{mm}}^{2}$)	0.	$1.\bar{3}$	$2.\bar{6}$	4.	0.	$2.25\bar{3}$	0.939866	2.182492	0.	1.2	2.52	3.852
$\left\langle d_{k}^{2}\right\rangle$ (${\mathrm{mm}}^{2}$)	2.	4.	6.	8.	2.	5.8	11.22	18.098	2.	4.	6.	8.
$\left\langle l_{k}\right\rangle$ (mm)	1.	2.	3.	4.	1.	2.	3.	4.	1.	2.	3.	4.
$\left\langle l_{k}^{2}\right\rangle$ (${\mathrm{mm}}^{2}$)	2.	6.	12.	20.	2.	6.	12.	20.	2.	6.	12.	20.

For higher scattering orders (k>4), we can still use Eqs. (25)–(31). As an example in Table 8, we show the deviation of the MC results for the moment $\left\langle d_{k}^{2}\right\rangle$ from Eq. (31) up to the tenth order for the different scattering functions considered. Except for one case, the deviation between MC results and reference RTE expected values is within two standard errors. These results confirm the same level of accuracy obtained in the other presented tables with the benchmarks used for k∈{1,…,4}. Similar results have also been obtained for the moments $\left\langle x_{k}^{2}\right\rangle$, $\left\langle y_{k}^{2}\right\rangle$, and $\left\langle z_{k}^{2}\right\rangle$ for the case of isotropic scattering [Eqs. (28) and (29)].

**Table 8:** Deviation of the MC results from the theory normalized to the MC standard error, $\frac{\langle d_{k}^{2}\rangle_{\mathrm{MC}}-\langle d_{k}^{2}\rangle_{\mathrm{RTE}}}{\sigma_{\langle d_{k}^{2}\rangle_{\mathrm{MC}}}}$, for the statistical moment $\left\langle d_{k}^{2}\right\rangle$ in a non-absorbing infinite medium with scattering coefficient $\mu_{s}=1\;{\mathrm{mm}}^{-1}$ for three different scattering functions: HG scattering function with g=0 and g=0.9, and Rayleigh scattering function. The number of simulated trajectories is $N=10^{10}$.

$\frac{\langle d_{k}^{2}\rangle_{\mathrm{MC}}-\langle d_{k}^{2}\rangle_{\mathrm{RTE}}}{\sigma_{\langle d_{k}^{2}\rangle_{\mathrm{MC}}}}$	$N=10^{10}$									
k	1	2	3	4	5	6	7	8	9	10
HG g=0	−0.150	−1.306	−0.931	−0.386	−0.785	−0.520	−0.312	−0.351	−0.756	−0.308
HG g=0.9	1.318	1.635	2.650	1.643	1.161	0.231	0.418	−0.017	0.239	0.379
Rayleigh	−0.117	−1.336	−0.687	−1.683	−1.548	−1.058	−1.212	−1.054	−1.057	−0.985

### 3.2 Second Step of the Verification (Sec. 2.2)

In this section, the radiance, the fluence rate, and the total mean path length spent inside a layered non-absorbing slab subjected to a Lambertian illumination calculated with MC simulations are compared with the exact analytical solutions of Sec. 2.2. This kind of verification is aimed to test the MC code regarding the effects of boundaries on photon migration. To this purpose, we have considered a layered slab geometry. In the considered examples, the boundary effects are tested both internally to the medium, betwen different layers, and also for the boundary with the external medium.

For this second step of the verification method, we have used Eqs. (33) and (37) for the radiance, Eqs. (34) and (38) for the fluence rate, and Eqs. (35) and (39) for the mean path lengths. We notice that, to the best of our knowledge, the methodical application of the radiance and the fluence rate for a verification procedure of MC codes is original. Radiance and fluence are fundamental quantities of transport theory and their correct calculation is crucial in many applications and also for the verification of MC codes.^40^^,^^41^

#### 3.2.1 Comparisons for the radiance

**Fig. 1:**
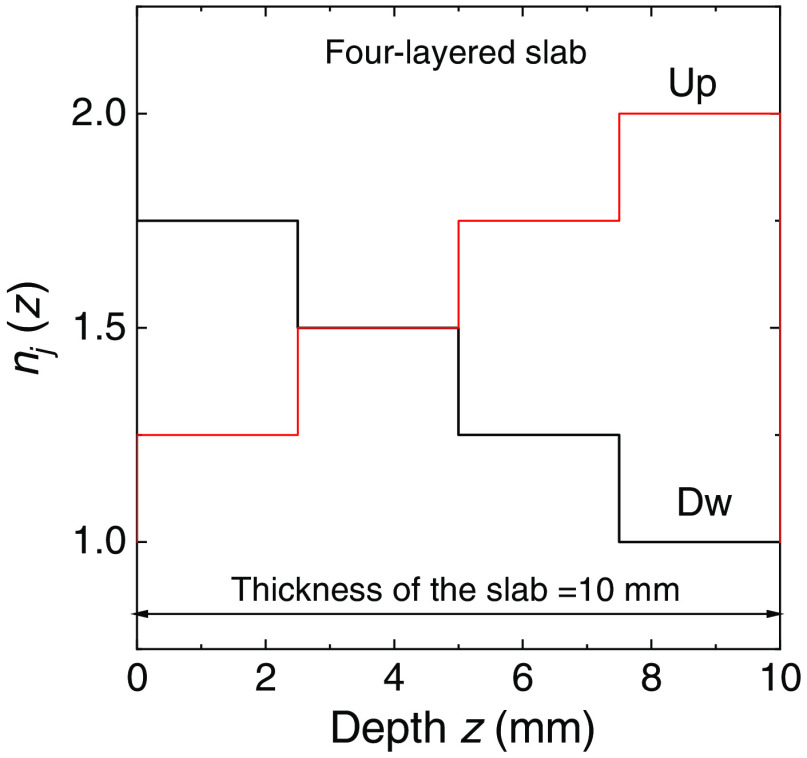
Profiles of the refractive index Up and Dw inside a four-layered slab of thickness 10 mm used in the MC simulations are shown in Fig. 2. The external refractive index is 1 for the profile Up and 2 for the profile Dw.

**Fig. 2:**
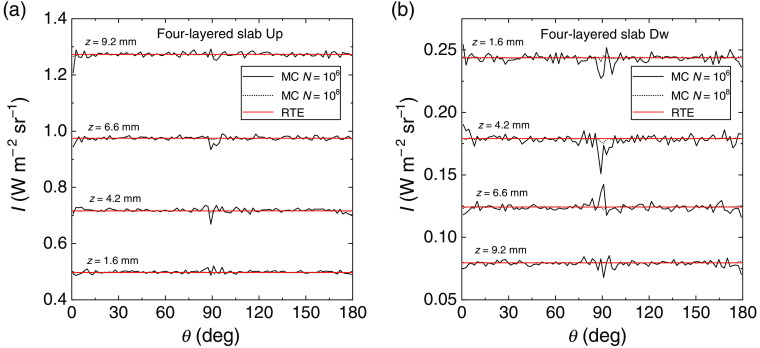
Radiance inside each layer of a non-absorbing four-layered slab of thickness 10 mm. Inside the slab the scattering coefficient is $\mu_{s}=1\;{\mathrm{mm}}^{-1}$ and the phase scattering function is obtained from the HG model with g=0. Two profiles of the refractive index inside the slab have been considered: Up and Dw, see Fig. 1. The external refractive index is 1 for the profile Up and 2 for the profile Dw. The solution of Eq. (33) (red curves) is compared with the results of MC simulations (noisy curves).

The MC simulations have been carried out assuming a unitary incoming flux. In the solutions given in Sec. 2.2, this is equivalent to assume in the theory $I_{0}=\frac{1}{\pi }$ $W{\;m}^{-2}\;{\mathrm{sr}}^{-1}$. All the layered slabs considered in this section are laterally infinitely extended and subjected to a uniform Lambertian illumination.^29^ The required uniform isotropic radiance illumination (Lambertian) of the slab can be carried out by using the intrinsic symmetries of this geometry that allow to replace the uniform illumination on the external surface by a single point illumination. This is possible by making use of the reciprocity theorem (or geometry equivalence) that allows to swap source and detector.^42^ Moreover, for the slab to have uniform illumination on both sides it is also needed to switch alternatively the illumination, i.e., the injection inside the slab of subsequent simulated photons, from one side to the other of the slab. As a first test for the verification of the radiance we considered a four-layered slab, where each layer was 2.5 mm thick and where we had two different distributions of the refractive index, defined as “Up” and “Dw.” These two distributions were characterized by an increasing and decreasing trend across the slab, respectively (Fig. 1). The external refractive index is 1 for the profile Up and 2 for the profile Dw. The scattering coefficient was fixed to $\mu_{s}=1\;{\mathrm{mm}}^{-1}$ in each layer of the slab and the scattering function was derived from the HG model with g=0. In Fig. 2, the radiance calculated with the MC code is compared to the solution of Eq. (33) for two numbers of simulated trajectories, i.e., $N=10^{6}$ and $10^{8}$. The radiance is shown in each layer. The convergence of the simulated radiance to the true values of the invariant solution can be clearly noted in each layer and for both distributions of the refractive index. In an error-free code, the improvement of the accuracy versus N can be also easily visualized for the radiance, that is isotropic and constant inside any sub-volume with a constant refractive index (see Sec. 2.2). This is shown in Fig. 2 where the fluctuations of the simulated radiance around the true values decrease for the larger N. In Fig. 2, it can also be noted the larger oscillations around 90 deg caused by the reduced sampling of photons around this angle.

**Fig. 3:**
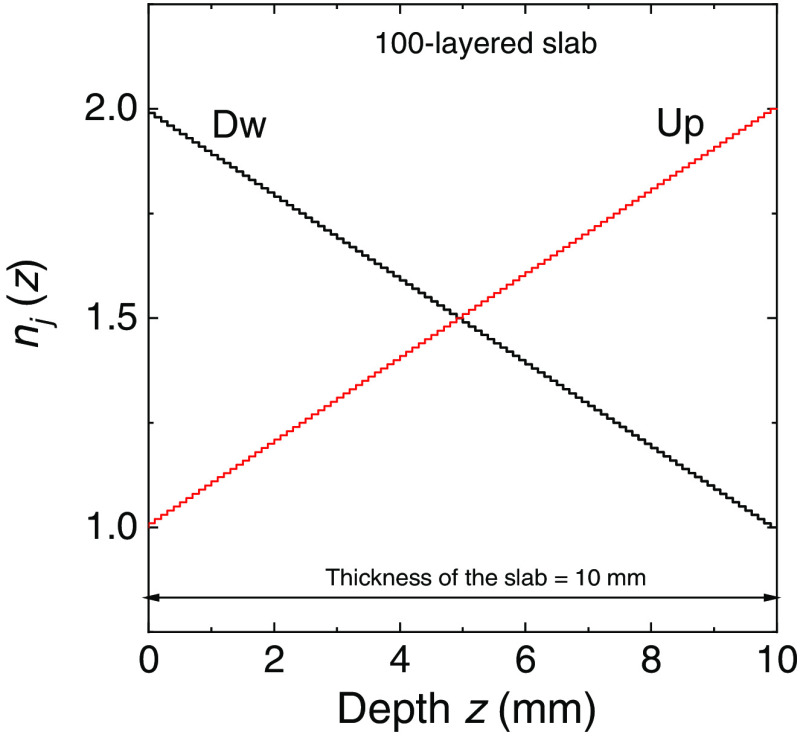
Profile of the refractive index Up and Dw inside 100-layered slab of 10 mm thickness used in the MC simulations shown in Figs. 4–9. The external refractive index is 1 for the profile Up and 2 for the profile Dw.

**Fig. 4:**
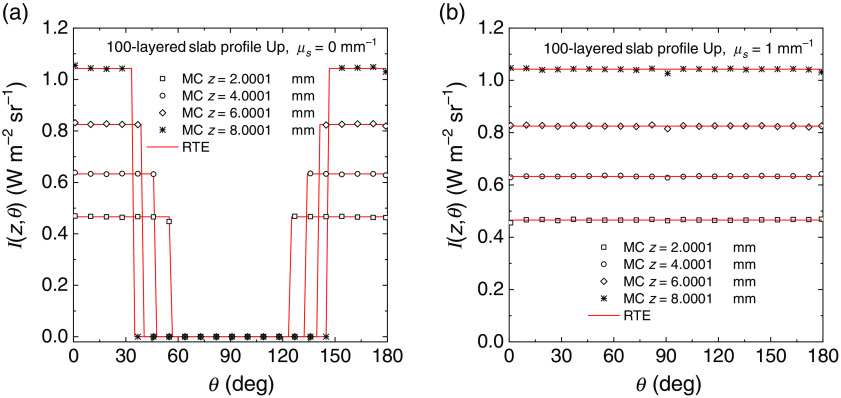
Radiance inside a non-absorbing 100-layered slab of thickness 10 mm calculated at different depths from the external boundary and for both the non-scattering (a) and scattering case (b). Inside the slab, we considered an HG phase scattering function with g=0. The profile of refractive index Up is considered, see Fig. 3. The solutions of Eq. (33) [red lines in (b)] and Eq. (37) [red lines in (a)] are compared with MC results (symbols).

**Fig. 5:**
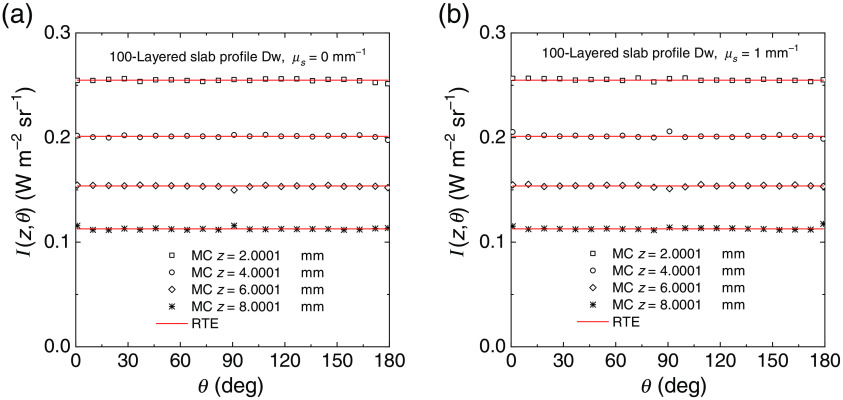
Radiance inside a non-absorbing 100-layered slab of thickness 10 mm calculated at different depths from the external boundary and for both the non-scattering (a) and scattering case (b). Inside the slab, we considered an HG phase scattering function with g=0. The profile of refractive index Dw is considered, see Fig. 3. The solutions of Eq. (33) [red lines in (b)] and Eq. (37) [red lines in (a)] are compared with MC results (symbols).

We have further tested the behavior of the radiance generated with the MC code by considering 100-layered slab, where each layer was 0.1 mm thick and where we had two different discrete distributions of the refractive index shown in Fig. 3 and denoted as Up, with an increasing refractive index from one side to the other, and Dw, with a decreasing refractive index from one side to the other. Also in this case the external refractive index is 1 for the profile Up and 2 for the profile Dw. For this 100-layered slab we have considered two values of the scattering coefficient: $\mu_{s}=0$ and $1\;{\mathrm{mm}}^{-1}$. The scattering function was the HG model with g=0. Figures. 4 and 5 show the comparisons of the radiance calculated with MC simulations and with Eqs. (33) ($\mu_{s}\neq 0$) and (37) ($\mu_{s}=0$) for the profiles Up and Dw, respectively. The results are shown for four selected depths z inside the slab, i.e., four selected layers, shown in the inset of the figures. For both refractive index profiles and scattering values, the MC simulations match the exact values of the RTE solutions. For the profile Up and $\mu_{s}=0\;{\mathrm{mm}}^{-1}$, we have the conditions of guided propagation and Eq. (33), i.e., $I_{j}=\frac{1}{\pi }{\left(\frac{n_{j}}{n_{e}}\right)}^{2}\;W\;m^{-2}\;{\mathrm{sr}}^{-1}$, for the radiance must be replaced by Eq. (37). In this case, the discontinuity of the radiance can be visualized for two values of the angle θ, as it is visible in Fig. 4. For incident angles greater than the maximum entrance angle for a given layer, the radiance drops to zero. This behavior is accurately reproduced by the MC results and is observed for all the depths considered. We can thus conclude that the calculations of radiance are widely verified with the exact solutions given in Sec. 2.2.

#### 3.2.2 Comparisons for the fluence rate

**Fig. 6:**
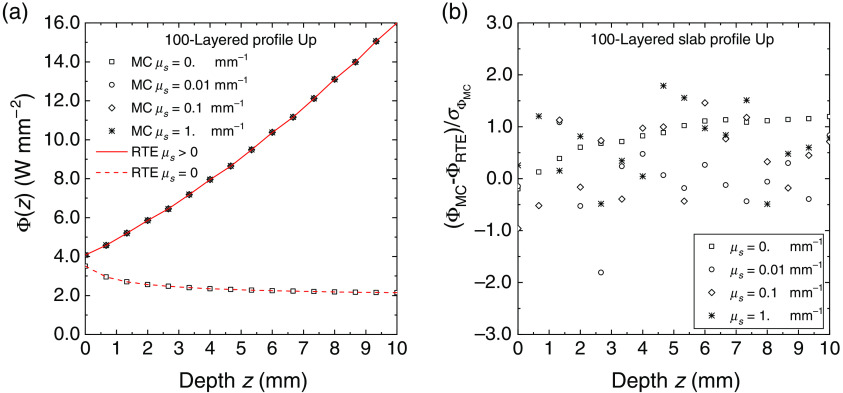
Fluence rate for the profiles Up inside 100-layered slab for several values of the scattering coefficient. (a) The results of MC simulations (symbols) and the RTE solution (continuous and broken lines). The fluence rate is plotted against the depth from the external surface of the slab. (b) The deviation between simulations and RTE solution normalized to the standard error.

**Fig. 7:**
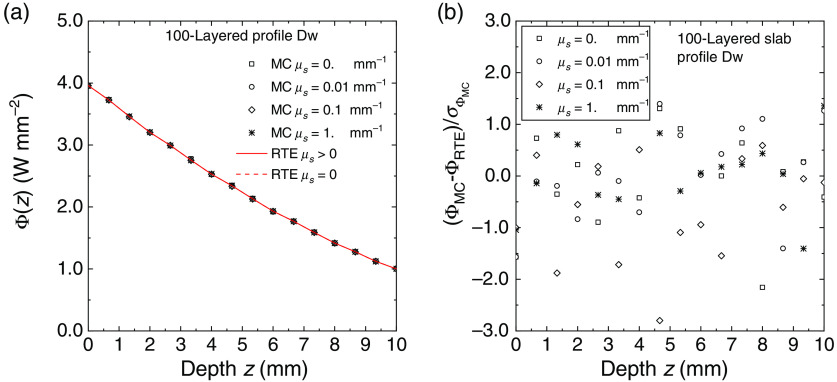
Fluence rate for the profiles Dw inside 100-layered slab for several values of the scattering coefficient. (a) The results of MC simulations (symbols) and the RTE solution (continuous and broken lines). The fluence rate is plotted against the depth from the external surface of the slab. (b) The deviation between the Monte Carlo simulations and the RTE solution normalized to the standard error.

In Figs. 6 and 7, for the same 100-layered slab used in the previous figures, we extended the verification to the fluence rate inside the slab for several values of the scattering coefficient and with a HG scattering function with g=0. The agreement between MC results and Eqs. (34) and (38) is excellent for all the values of the scattering coefficient and for both the refractive index distributions Up and Dw. The figures also show (right panels) the deviation between MC results and the RTE solutions which is within about two standard errors of the calculated fluence. The MC results have been reported for a subset of 100 layers to assure a clear reading of the symbols versus the depth z inside the slab. For the profile Up there is a discontinuity of the fluence rate between the scattering and non-scattering cases: the fluence switches from the value given by the invariance property [Eq. (34)], i.e., $\Phi_{j}=4(\frac{n_{j}}{n_{e}}{)}^{2}\;W\;m^{-2}$, to the value obtained with Eq. (38). In Fig. 7, the two RTE curves for $\mu_{s}>0$ and $\mu_{s}=0$ are indistinguishable. We can conclude that also the calculations of fluence rate are very well verified with the exact solutions given in Sec. 2.2.

#### 3.2.3 Comparisons for the mean path length

**Fig. 8:**
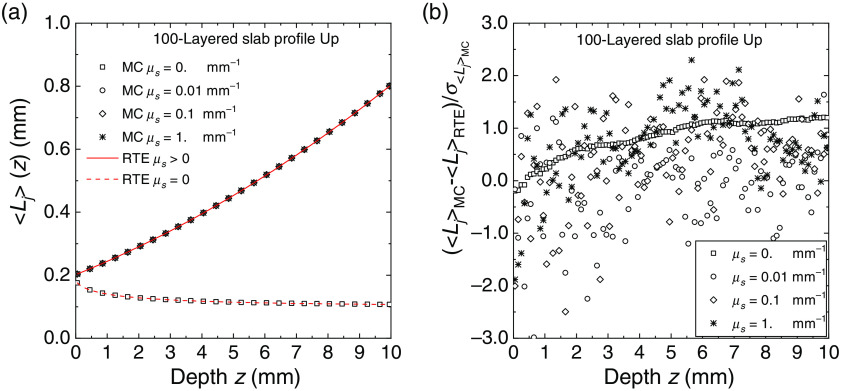
Average internal path length $\left\langle L_{j}\right\rangle$ spent in the layers of 100-layered slab for the profiles Up for several values of the scattering coefficient. (a) The results of MC simulations (symbols) and the RTE solution (continuous and broken lines). The path length is plotted against the depth from the external surface of the slab. (b) The deviation between the Monte Carlo simulations and the RTE solution normalized to the standard error.

**Fig. 9:**
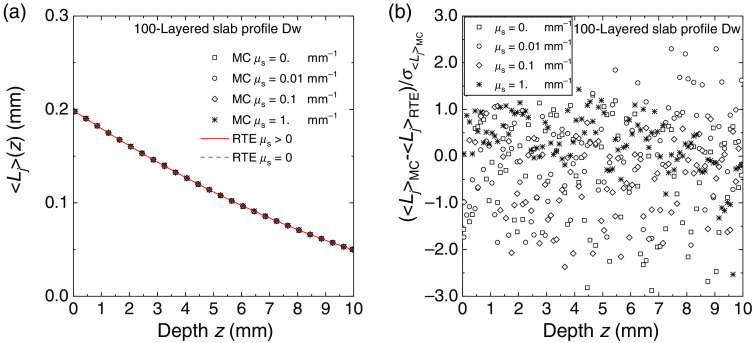
Average internal path length $\left\langle L_{j}\right\rangle$ spent in the layers of 100-layered slab for the profiles Dw for several values of the scattering coefficient. (a) The results of MC simulations (symbols) and the RTE solution (continuous and broken lines). The path length is plotted against the depth from the external surface of the slab. (b) The deviation between simulations and RTE solution normalized to the standard error.

In Figs. 8 and 9, for the same 100-layered slab used for generating Figs. 4–7, we compared the partial mean path length $\left\langle L_{j}\right\rangle$ spent inside the layers of the slab calculated with MC simulations and with Eqs. (35) and (39). The agreement between MC and exact solutions is excellent and very similar to that obtained for the fluence rate in the previous figures. For the profile Up, we also have a discontinuity of $\left\langle L_{j}\right\rangle$ between $\mu_{s}\neq 0$ and $\mu_{s}=0$: $\left\langle L_{j}\right\rangle$ switches from the value given by the invariance property [Eq. (35)], i.e., $\langle L_{j}\rangle =2s_{j}(\frac{n_{j}}{n_{e}}{)}^{2}$ ($s_{j}$ thickness of the layer j, in this case, $s_{j}=0.1\;\mathrm{mm}$), to the value obtained with Eq. (39). In Fig. 9, the two RTE curves for $\mu_{s}>0$ and $\mu_{s}=0$ are indistinguishable. The deviation between MC results and RTE solutions is also in this case within about two standard errors. We have thus evidence that also the calculations of the mean path length are widely verified with the exact solutions given in Sec. 2.2.

## 4 Discussion

In the proposed two-step verification method, we have exploited the complementary characteristics of two kinds of benchmarks: one set of analytical benchmarks sensitive to the single-scattering properties in an infinite medium and another set sensitive to the effects of boundaries. Therefore, the two steps are useful to test an MC code for the correctness of trajectories extraction (first step) and the correctness of photons intersection with boundaries, including the calculations of partial and total path lengths (second step). The proposed method significantly extends the verification methods previously published in Refs. 2 and 3. In fact, the case of isotropic scattering in an infinite medium can now be applied for any scattering order [Eqs. (28)–(31)]. Moreover, the benchmark proposed in Martelli et al.,^3^ which specifically focused on the calculation of partial and total mean path lengths, now also includes other fundamental radiometric quantities such as radiance and fluence rate.

The presented results show an increasing accuracy with the number of simulated trajectories, which is limited only by machine precision. In practice, the results and the related t-tests confirm the reliability of our MC code and serve as an example of the proposed approach.

Probably, at this point, it is not useless to repeat the following concept: the success of a test (i.e., a null hypothesis not rejected) does not guarantee that a particular MC code performs correctly for all the cases envisioned. Only the failure of the test gives the certainty of having an error inside the code.^1^ Thus, the careful reader should realize that, from a practical point of view, no existing method allows us to detect 100% of the errors in an MC code. Nevertheless, we believe that our proposed method is a valuable contribution to increasing the sensitivity of a test to detect coding errors. Also, the two-step method allows one to be more specific about the origin of the error, i.e., if it is caused by an incorrect implementation of the phase function and trajectories’ extraction (step 1) or by an incorrect treatment of the boundaries (step 2). Thus, in this frame, the present contribution certainly represents a fundamental improvement.

Another observation is about testing for absorption effects. Biological tissues are absorbing media and the absorption coefficient is always considered in MC simulations. We notice that even if the two-step method involves only non-absorbing media, this fact does not compromise the generality of the verification. In fact, absorption does not change the photons’ trajectories and its effect can be accounted for with a straightforward implementation of the mBLL,^28^ without any computational criticality. At the core of the application of the mBLL is the correct evaluation of the partial path lengths, which is tested in step 2 of our method.

One final observation is about the extension of step 2. In the examples reported in this work, we simplified the application of the second step by using the symmetries of the media considered, which allowed us to implement the Lambertian illumination at one point of the external boundary and use the reciprocity theorem to calculate the quantities of interest. However, we stress that the proposed method can be applied to any complex geometry where a full Lambertian illumination can be implemented. This would allow one to test an MC code (for the boundary effects) directly for more realistic cases of interest, without resorting to simplifying the assumptions on the geometry and the distribution of the scattering properties. Future research will be directed to verify the feasibility of this point.

## 5 Appendix: Moments for Isotropic Scattering Functions

In this appendix, we prove the validity of Eqs. (28)–(31) in Sec. 2.1.2. Equation (31) can be derived with an exact procedure from the RTE according to the results of Ref. 32. A separate proof must be given for Eqs. (28)–(30). We observe that the distribution function of the random variable $z_{k}^{2}$, due to the pencil beam source emitting along z and to the hypothesis of isotropic scattering, differs from the distributions of $x_{k}^{2}$ and $y_{k}^{2}$ by the first scattering order. After the first scattering, due to the isotropic distribution of the scattering angle, the two distributions are indistinguishable. For the first scattering order, both distributions of $x_{k}^{2}$ and $y_{k}^{2}$ are null. This fact implies that the difference between $\left\langle z_{k}^{2}\right\rangle$ and $\left\langle x_{k}^{2}\right\rangle$ is given by the right term of Eq. (4). Let’s verify this property. The random variable $z_{k}$ can be written as

$$z_{k}=z_{1}+(z_{k}-z_{1})=z_{1}+\Delta z_{k}.$$ (40)

Therefore,

$$z_{k}^{2}=z_{1}^{2}+\Delta z_{k}^{2}+2z_{1}\Delta z_{k}.$$ (41)

We note that the random variables $z_{1}$ and $\Delta z_{k}$ are independent and also that $\langle \Delta z_{k}\rangle =0$ because of the hypothesis of isotropic scattering. Thus, taking the average of the above equation, we get

$$\langle z_{k}^{2}\rangle =\langle z_{1}^{2}\rangle +\langle \Delta z_{k}^{2}\rangle +2\langle z_{1}\rangle \langle \Delta z_{k}\rangle =\langle z_{1}^{2}\rangle +\langle \Delta z_{k}^{2}\rangle .$$ (42)

And, because of the isotropic scattering, the random variables $\Delta z_{k}$, $x_{k}$, and $y_{k}$ have the same distribution function. Thus we have

$$\langle z_{k}^{2}\rangle =\langle x_{k}^{2}\rangle +\frac{2}{\mu_{s}^{2}}=\langle y_{k}^{2}\rangle +\frac{2}{\mu_{s}^{2}}.$$ (43)

By exploiting Eqs. (31) and (43), we can write

$$3\langle x_{k}^{2}\rangle +\frac{2}{\mu_{s}^{2}}=\frac{2k}{\mu_{s}^{2}},$$ (44)

from which we obtain

$$\langle x_{k}^{2}\rangle =\langle y_{k}^{2}\rangle =\frac{2}{3\mu_{s}^{2}}(k-1),$$ (45)

which proves Eq. (28), and

$$\langle z_{k}^{2}\rangle =\frac{2}{3\mu_{s}^{2}}(k+2),$$ (46)

which proves Eq. (29). Finally, Eq. (30) is a trivial consequence of the above relations. Thus, all the relations given in Sec. 2.1.2 for isotropic scattering are exact within the RTE.
